# Metals Exposures of Residents Living Near the Akaki River in Addis Ababa, Ethiopia: A Cross-Sectional Study

**DOI:** 10.1155/2015/935297

**Published:** 2015-11-22

**Authors:** Ellen Yard, Tesfaye Bayleyegn, Almaz Abebe, Andualem Mekonnen, Matthew Murphy, Kathleen L. Caldwell, Richard Luce, Danielle Rentz Hunt, Kirubel Tesfaye, Moa Abate, Tsigereda Assefa, Firehiwot Abera, Kifle Habte, Feyissa Chala, Lauren Lewis, Amha Kebede

**Affiliations:** ^1^Centers for Disease Control and Prevention, National Center for Environmental Health, Atlanta, GA 30341, USA; ^2^Centers for Disease Control and Prevention, Epidemic Intelligence Service, Atlanta, GA 30333, USA; ^3^Ethiopian Public Health Institute, Addis Ababa, Ethiopia; ^4^Centers for Disease Control and Prevention, Center for Global Health, Atlanta, GA 30333, USA; ^5^Abt Associates Inc., Atlanta, GA 30345, USA

## Abstract

*Background*. The Akaki River in Ethiopia has been found to contain elevated levels of several metals. Our objectives were to characterize metals exposures of residents living near the Akaki River and to assess metal levels in their drinking water.* Methods*. In 2011, we conducted a cross-sectional study of 101 households in Akaki-Kality subcity (near the Akaki River) and 50 households in Yeka subcity (distant to the Akaki River). One willing adult in each household provided urine, blood, and drinking water sample.* Results*. Urinary molybdenum (*p* < 0.001), tungsten (*p* < 0.001), lead (*p* < 0.001), uranium (*p* < 0.001), and mercury (*p* = 0.049) were higher in Akaki-Kality participants compared to Yeka participants. Participants in both subcities had low urinary iodine; 45% met the World Health Organization (WHO) classification for being at risk of moderate iodine deficiency. In Yeka, 47% of households exceeded the WHO aesthetic-based reference value for manganese; in Akaki-Kality, only 2% of households exceeded this value (*p* < 0.001). There was no correlation between metals levels in water samples and clinical specimens.* Conclusions*. Most of the exposures found during this investigation seem unlikely to cause acute health effects based on known toxic thresholds. However, toxicity data for many of these metals are very limited.

## 1. Introduction

**Figure 1:**
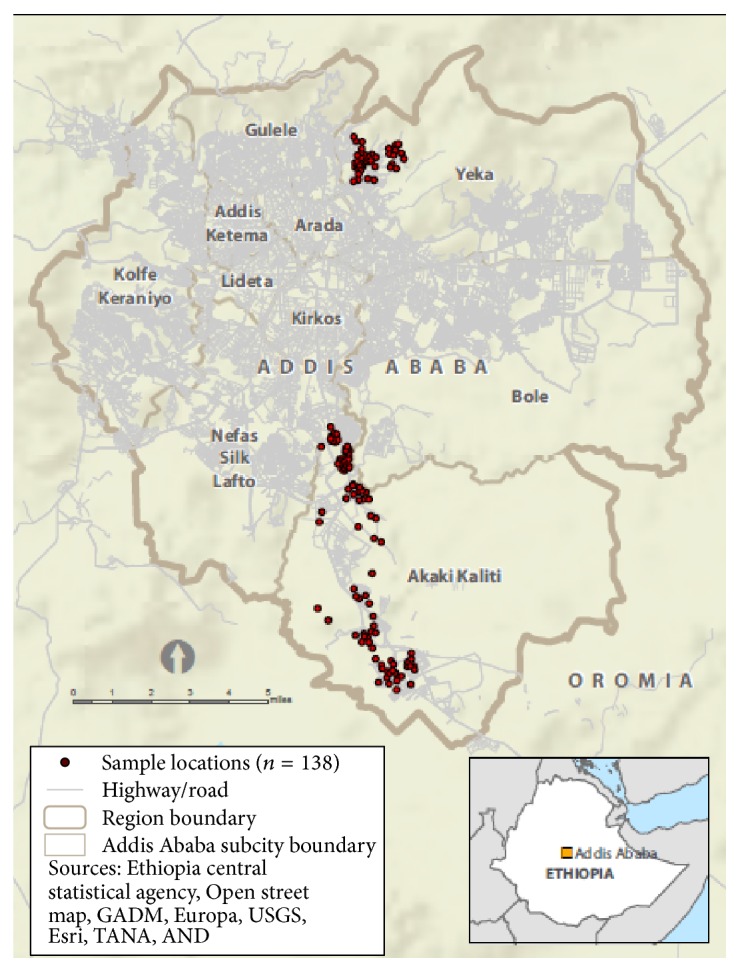
Sampling locations—Addis Ababa, Ethiopia, 2011.

The Akaki River in Ethiopia is purported to be contaminated with multiple toxic agents, including metals. The river runs through residential, commercial, and industrial areas of the capital city of Addis Ababa (Figure 1), where it can be subjected to industrial (e.g., tanneries and battery factories) [1–3] and human (e.g., domestic wastewater and surface run-off) [1] pollutants. Previous studies describe metal levels in the river itself [1], as well as nearby fish [4], soil [1], and irrigated vegetables [1, 5]. One study found that manganese and iron levels in the Akaki River exceeded limits set by the Ethiopia Environmental Protection Agency. This study also found measurable amounts of cadmium, chromium, copper, zinc, manganese, iron, and nickel in soil and vegetables irrigated by the Akaki River, with all vegetables having chromium levels that exceeded the maximum limit [1]. Another study found that vegetables irrigated by the Akaki River had levels of cadmium and lead that exceeded the maximum limit [5].

There is little information in the published literature of what effect, if any, the Akaki River might have on residents living downstream from Addis Ababa, and whether residents living in this area might have increased risk of exposure to metals and other contaminants compared to the general Ethiopian population. It is possible that these residents could use the river water for irrigation, animal husbandry, or other uses [1, 2].

Our objectives were to characterize metal exposures in urine, blood, and drinking water samples of a community living near the Akaki River and to compare these levels to a community living distant to the Akaki River. We chose Akaki-Kality subcity as our community of interest near the Akaki River. Akaki-Kality is 25 kilometers south of Addis Ababa, and the Akaki River flows through western Akaki-Kality after passing through Addis Ababa. Approximately 200,000 people reside in Akaki-Kality, which is 125 square kilometers and contains over half of the industries of Addis Ababa [6]. We chose Yeka subcity as our community of interest distant to the Akaki River. Approximately 350,000 people reside in Yeka subcity, which is 86 square kilometers and situated 10 kilometers east of central Addis Ababa.

## 2. Methods

### 2.1. Study Design and Sampling

We conducted a cross-sectional study of 101 houses in Akaki-Kality and 50 houses in Yeka from June 28 to July 1, 2011. We first stratified participating subcities into woredas (i.e., districts). In Akaki-Kality, six of the eight woredas bordering the Akaki River agreed to participate; in Yeka, two of the thirteen woredas agreed to participate. Field teams sampled households systematically within each participating woreda by randomly selecting a starting point and systematically select every *n*th housing unit. We calculated “*n*” separately for each subcity, by taking the total estimated population of the participating area (Akaki-Kality: 10,548; and Yeka: 8,985) and dividing by the target sample size (Akaki-Kality: 100 and Yeka: 50, resp.). Thus, we selected 1 in every 105 houses in Akaki-Kality and 1 in every 180 houses in Yeka. If a household did not answer the door or refused to participate, then the field team solicited participation from the next closest household.

### 2.2. Data Collection

All field teams were trained in the study methods and interviewing techniques immediately prior to the field work. Field teams did the following: obtained informed consent from the head of household; collected a drinking water sample; conducted a household-level questionnaire (e.g., household demographics and sources of food and drinking water); collected urine and blood sample from one volunteer; and conducted an individual-level questionnaire from the same volunteer (e.g., occupation, whether they drank alcohol, and health status). All interviews were conducted in Amharic.

### 2.3. Laboratory Analysis

The US Centers for Disease Control and Prevention (CDC), Division of Laboratory Sciences in Atlanta, Georgia, analyzed urine metals (antimony, arsenic, barium, beryllium, cadmium, cesium, chromium, cobalt, iodine, lead, mercury, molybdenum, nickel, platinum, thallium, tungsten, and uranium) and blood metals (cadmium, lead, manganese, mercury, and selenium) using inductively coupled dynamic reaction cell plasma mass spectrometry (ICP-DRC-MS) and sector field inductively coupled plasma mass spectrometry (SF-ICP-MS; Table 1) [7]. This list of metals was chosen because it is a comprehensive list of the most common metals that persons might be exposed to, and comparison data for these metals exist for the US population.

Spot urine samples can vary in dilution between persons; thus, we adjusted all urine samples for creatinine to help account for this dilution and to facilitate comparisons between participants. We present most urine metals in units of *μ*g/g creatinine and most blood metals in units of *μ*g/L; the exceptions were urinary iodine and blood lead, which are commonly reported as *μ*g/L and *μ*g/dL, respectively.

The Environmental Science Corporation (ESC) Lab Sciences in Nashville, Tennessee, analyzed drinking water samples for nitrates and metals using the US Environmental Protection Agency's (EPA) method 200.7 for chromium, cobalt, copper, manganese, and nickel; EPA method 200.8 for arsenic, cadmium, and lead; EPA method 245.1 for mercury; and EPA method 353.2 for nitrates.

### 2.4. Data Analysis

We analyzed data using SAS version 9.2. We used maximum likelihood estimation to calculate geometric means and to compare metal concentrations by subgroup. This method accounts for the left censoring that results when some metal concentrations are below the limit of detection (LOD). We considered *p* values <0.05 to be statistically significant.

At the time of this investigation, there was little information available regarding baseline metal levels in the general Ethiopian and other East African populations. Thus, to put our results into perspective, we compared urine and blood metal concentrations among study participants to the US population, as calculated through the US National Health and Nutrition Examination Survey (NHANES) [8]. We compared drinking water concentrations among study participants to the guideline values established by the World Health Organization (WHO) [9].

For urine or blood metals in which at least half of the study population had detectable levels, we conducted multivariable linear regression. In these models, the log-transformed metal was the outcome, and characteristics were considered for inclusion as independent variables in the model if they were statistically significant in univariable analyses.

This investigation was initiated following a request for CDC Epi-Aid assistance from the Ethiopia Federal Ministry of Health to collect baseline information on exposure to possibly harmful contaminants in water from the Akaki River.

## 3. Results

### 3.1. Study Participation

Overall, 90% of households were at home at the time of the visit, and 76% of households that were at home participated. The completed sample size was 101 in Akaki-Kality and 50 in Yeka. Households were clustered tightly together in Yeka, and in Akaki-Kality households were located along the Akaki River (Figure 1).

### 3.2. Questionnaire

Household members in both subcities ranged in age from 1 to 80 years, with a mean of 28 years in Akaki-Kality and 26 years in Yeka. Approximately half (53%) were female. Body mass index, amount of time living in the subcity, occupation, health behaviors, and drinking water characteristics did not differ substantially between Akaki-Kality and Yeka (Table 2). Body mass index ranged from 14 to 38 kg/m^2^, with a median of 22 kg/m^2^. Participants had lived in their subcity for an average of 18 years. One-third (38%) did not have an occupation, 17% worked in a factory or as a laborer, and 12% worked in business. Few participants reported drinking alcohol (17%), chewing khat (5%), or smoking cigarettes (2%).

**Figure 2:**
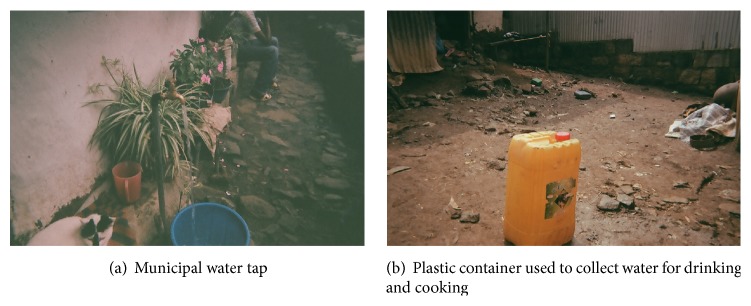
Photos of drinking water from participant homes—Addis Ababa, Ethiopia, 2011.

All participating households had municipality-provided tap water for drinking. This tap was usually in the yard, and water was collected in a plastic container (Figure 2). In Akaki-Kality, the municipal supply was drawn from groundwater; in Yeka, the municipal supply was drawn from surface water.

One-fourth of participants were concerned that their tap water could cause health problems; this was more common in Yeka (34%) compared to Akaki-Kality (18%) (*p* = 0.02). Some participants felt insecure about their water's cleanliness (34%), or they noted that their water had a bad color (17%), bad taste (9%), or bad smell (5%). These concerns did not differ by subcity. It was these tap water samples that were collected as drinking water samples.

### 3.3. Urine and Blood Metals

Table 3 depicts urine and blood metal concentrations by subcity. Urinary molybdenum (*p* < 0.001), tungsten (*p* < 0.001), lead (*p* < 0.001), uranium (*p* < 0.001), and mercury (*p* = 0.049) were higher in Akaki-Kality participants compared to Yeka participants; Yeka participants had higher concentrations of urinary arsenic (*p* = 0.009). Compared to the US adult (aged ≥ 20 years) population in 2011-2012, study participants had mean levels of urinary cobalt that were over four times higher and mean levels of urinary molybdenum that were over two times higher. Conversely, urinary cesium and mercury levels were considerably lower in our participants compared to the US, with mean levels being over two times higher in the US.

Median urinary iodine concentrations were relatively low in both subcities (Akaki-Kality: 53.9 *μ*g/L, Yeka: 60.1 *μ*g/L). As a result, both subcities met the World Health Organization's (WHO) guidelines as having moderate iodine deficiency (population median iodine levels from 50 to 99 *μ*g/L) [10].

### 3.4. Drinking Water Analytes

Table 4 depicts water analyte concentrations by subcity. In Yeka, 47% of households exceeded the WHO aesthetic-based reference value for manganese. In Akaki-Kality, only 2% of households exceeded the reference value (*p* < 0.001).

Most drinking water samples contained detectable levels of nitrates (81%); mean nitrate concentrations were higher in Akaki-Kality (3.5 mg/L) compared to Yeka (0.06 mg/L) (*p* < 0.001). One drinking water sample exceeded the WHO nitrates reference value of 50 mg/L. This sample was from Yeka and had a very high nitrates concentration (740 mg/L). Despite this, the household did not report any specific problems with their water.

The majority of drinking water samples did not contain detectable amounts of lead, copper, cadmium, arsenic, chromium, cobalt, or mercury, and none of these levels exceeded reference values.

### 3.5. Risk Factors

We examined whether metal exposures varied by occupation, diet, and smoking and alcohol use. Compared to other participants, factory workers had higher concentrations of urinary arsenic (GM = 7.46 versus 5.60 *μ*g/g creatinine, *p* = 0.029) and urinary nickel (GM = 11.5 versus 8.34 *μ*g/g creatinine, *p* = 0.012).

Participants who reported drinking alcohol had lower levels of urinary cobalt (GM = 1.13 *μ*g/g creatinine) compared to participants who did not report drinking alcohol (GM = 1.67 *μ*g/g creatinine, *p* < 0.001). Participants who chewed khat had lower concentrations of urinary cobalt (GM = 1.06 *μ*g/g creatinine versus GM = 1.58 *μ*g/g creatinine; *p* = 0.048) and urinary tungsten (GM = 0.19 *μ*g/g creatinine versus 0.11 *μ*g/g creatinine; *p* = 0.038) compared to those who did not chew khat. There were no correlations between metal levels in drinking water and urine or blood, and no significant associations were found in multivariable analyses (results not shown).

## 4. Discussion

We assessed urinary and blood metals in a subset of the Ethiopian population, which provides data that have previously been unavailable. Participants had exposure to a range of metals, some of which were elevated compared to a US reference population. Most of the urine and blood metal levels found during this investigation seem unlikely to cause acute health effects based on known toxic thresholds. However, toxicity data for many of these metals are very limited, and the risk of chronic health effects from possible long-term exposures to these metals is not fully known and would likely depend on other factors such as concurrent exposures and other health risks.

Urinary cobalt and molybdenum were substantially higher in our participants compared to the US population. This could be a result of a variety of potential environmental exposures. Cobalt is a naturally occurring element that is found in foods such as fish, cocoa, bran, molasses, and some green leafy vegetables. It is also used in alloys (mixtures of metals) and as a drier for paint and porcelain enamel. People are exposed to cobalt through food, water, and air. Additionally, higher exposures can occur in industrial occupations that utilize cobalt. It is unknown if any of the industries near the Akaki River were utilizing cobalt. Cobalt exposure has been associated with allergic contact dermatitis [11], occupational asthma [12], interstitial lung disorder [13], and cardiomyopathy [14], and animal studies suggest it may be a potential carcinogen. However, there are limited data relating urinary cobalt concentrations to health effects, and no validated, consensus measurement for the minimal level associated with toxic effects in an environmental, nonoccupational setting. Furthermore, health outcomes can depend on the route of exposure.

Molybdenum is an essential trace element, often consumed through the diet and drinking water. There is limited information concerning adverse health effects, and human toxicity is considered to be low [8]. Chronic exposure to molybdenum levels in the range of 0.14–0.21 mg/kg per day has been associated with a gout-like illness [15, 16]. There are limited data relating urinary molybdenum concentrations to these specific health effects.

This investigation was initiated based on concerns that the Akaki River might be causing elevated levels of metals exposures in nearby communities. Even though we found several differences in urine metal concentrations between Akaki-Kality and Yeka participants, we did not find any evidence that exposures were a result of proximity to the Akaki River or of domestic use of the river water. Participants were not using the Akaki River for drinking, cooking, or bathing, and we did not detect metals in most drinking water samples. It is possible that the differing metal profiles between Akaki-Kality and Yeka participants are a result of other sources of metals exposure, such as differences in the surrounding air quality or differing dietary habits.

We found that participants who reported growing their own vegetables had lower lead levels compared to participants who did not grow their own vegetables. This could be a spurious correlation, particularly given that there were no differences in other metals. We do not know the exact source of vegetables that were not homegrown, nor do we know the source of the water that they were irrigated with. However, given that a prior study found lead in vegetables irrigated by the Akaki River [5], it is possible that vegetables that are not homegrown are purchased from one of the nearby markets that are using the Akaki River for irrigation.

Many Akaki-Kality and Yeka participants had low iodine concentrations. Iodine is an essential nutrient for newborns, particularly from the second trimester of pregnancy through the third year of life. It stimulates the thyroid gland to produce thyroid hormone, which in turn promotes brain development. Iodine deficiency can cause impaired mental function, goiter, and hypothyroidism, and it is considered to be an important cause of preventable brain damage [10, 17]. According to the World Health Organization, median iodine concentrations ranging from 50 to 99 *μ*g/L indicate that a population may have mild iodine deficiency [10]. We found low median urine iodine concentrations in both subcities—only 53.9 *μ*g/L in Akaki-Kality and 60.1 *μ*g/L in Yeka. This is presumably due to a lack of iodized salt [10]. Iodine deficiency is relatively easy to treat. In most populations, people obtain sufficient iodine by consuming iodine-fortified salt [10]. Ethiopia had achieved almost universal salt iodization by the early 1990s. However, after Eritrea seceded from Ethiopia, Ethiopia's iodized salt consumption dropped to 5% [18]. Our findings suggest that continued work is needed on improving access to iodized salt in Ethiopia.

Our investigation had several limitations. Although the motive for study was the Akaki River, the study itself only evaluated people and household drinking water, and thus exposures cannot be attributed directly to the river. There are no good data on baseline metal exposures in Ethiopia, and thus we cannot compare the metal profiles of our participants to the underlying population. Because this was a one-time, cross-sectional assessment, it is possible we could have missed any seasonal differences or sporadic exposures, particularly because the half-life of some metals in the body can be hours to days. Our sampling was representative of the general Akaki-Kality population; it is possible that there could be specific segments of Akaki-Kality or other subcities that might have had greater metals exposures.

Continuing to perform biomonitoring investigations in Ethiopia and other countries might help public health professionals identify regions with unique risks to specific exposures. Biomonitoring can also help monitor population-level exposures over time in order to determine the impact of changes to the surrounding area.

## Figures and Tables

**Table:** (a) Urine

Analyte	*n*	LOD	% >LOD	Analytic technique
Antimony	147	0.032 *µ*g/L	57%	ICP-DRC-MS
Arsenic	136	1.25 *µ*g/L	97%	ICP-DRC-MS
Barium	147	0.12 *µ*g/L	100%	ICP-DRC-MS
Beryllium	147	0.072 *µ*g/L	2%	ICP-DRC-MS
Cadmium	147	0.042 *µ*g/L	88%	ICP-DRC-MS
Cesium	147	0.066 *µ*g/L	99%	ICP-DRC-MS
Chromium	147	0.105 *µ*g/L	95%	SF-ICP-MS
Cobalt	147	0.041 *µ*g/L	100%	ICP-DRC-MS
Iodine	147	1.4 *µ*g/L	100%	ICP-DRC-MS
Lead	147	0.10 *µ*g/L	99%	ICP-DRC-MS
Mercury	141	0.05 *µ*g/L	80%	ICP-DRC-MS
Molybdenum	147	0.92 *µ*g/L	100%	ICP-DRC-MS
Nickel	147	0.337 *µ*g/L	100%	SF-ICP-MS
Platinum	147	0.009 *µ*g/L	3%	ICP-DRC-MS
Thallium	147	0.015 *µ*g/L	100%	ICP-DRC-MS
Tungsten	147	0.021 *µ*g/L	95%	ICP-DRC-MS
Uranium	147	0.0017 *µ*g/L	93%	ICP-DRC-MS

**Table:** (b) Blood

Analyte	*n*	LOD	% >LOD	Analytic technique
Cadmium	132	0.16 *µ*g/L	88%	ICP-DRC-MS
Lead	132	0.25 *µ*g/L	100%	ICP-DRC-MS
Manganese	132	1.06 *µ*g/L	100%	ICP-DRC-MS
Mercury	132	0.16 *µ*g/L	25%	ICP-DRC-MS
Selenium	132	30 *µ*g/L	100%	ICP-DRC-MS

**Table:** (c) Drinking water

Analyte	*n*	LOD	% >LOD	Analytic technique	Comparison Value (CV)^a^
Arsenic	143	0.001 mg/L	1%	ICP-MS	0.01 mg/L
Cadmium	143	0.0005 mg/L	1%	ICP-MS	0.003 mg/L
Chromium	143	0.010 mg/L	0%	ICP-AES	0.05 mg/L
Cobalt	143	0.010 mg/L	0%	ICP-AES	N/A
Copper	143	0.020 mg/L	3%	ICP-AES	2 mg/L
Lead	143	0.001 mg/L	18%	ICP-MS	0.01 mg/L
Manganese	143	0.010 mg/L	27%	ICP-AES	N/A
Mercury	143	0.0002 mg/L	0%	CVAAS	0.006 mg/L
Nitrates	150	0.10 mg/L	81%	FIA	50 mg/L

LOD = limit of detection; ICP-DRC-MS = inductively coupled dynamic reaction cell plasma mass spectrometry; ICP-MS = inductively coupled plasma mass spectrometry; SF-ICP-MS = sector field inductively coupled plasma mass spectrometry; ICP-AES = inductively coupled plasma atomic emission spectroscopy; CVAAS = cold vapor atomic absorption spectrometry; FIA = Flow Injection Analysis.

^a^Comparison values determined using the World Health Organization guideline values for water.

**Table 2:** Participant characteristics, by subcity—Addis Ababa, Ethiopia, 2011.

	Overall (*n* = 151)	Akaki-Kality (*n* = 101)	Yeka (*n* = 50)
Body mass index (kg/m^2^; *n* = 75)			
Range	14–38	14–38	18–35
Mean (St. dev.)	23 (4)	23 (4)	23 (4)
Median	22	22	23
Years living in subcity (*n* = 141)			
Range	1–50	1–50	1–35
Mean (St. dev.)	18 (11)	18 (12)	18 (10)
Median	19	18	20
Occupation (*n* = 132)			
None	50 (38%)	35 (39%)	15 (35%)
Factory/laborer	23 (17%)	17 (19%)	6 (14%)
Business	16 (12%)	12 (13%)	4 (9%)
Student	15 (11%)	11 (12%)	4 (9%)
Teacher/secretary	8 (6%)	5 (6%)	3 (7%)
Other^a^	20 (15%)	9 (10%)	11 (26%)
Drink alcohol (*n* = 144)			
Yes	24 (17%)	19 (19%)	5 (11%)
No	120 (83%)	79 (81%)	41 (89%)
Chew khat^*∗*^ (*n* = 149)			
Yes	7 (5%)	2 (2%)	5 (10%)
No	142 (95%)	98 (98%)	44 (90%)
Smoke cigarettes (*n* = 148)			
Yes	3 (2%)	1 (1%)	2 (4%)
No	145 (98%)	99 (99%)	46 (96%)
Eat homegrown vegetables (*n* = 151)			
Yes	18 (12%)	9 (9%)	9 (18%)
No	133 (88%)	92 (91%)	41 (82%)
Add chlorine to drinking water (*n* = 151)			
Yes	12 (8%)	7 (7%)	5 (10%)
No	139 (92%)	94 (93%)	45 (90%)
Drinking water characteristics (*n* = 151)			
Cloudy/muddy	26 (17%)	19 (19%)	7 (14%)
Bad taste	13 (9%)	10 (10%)	3 (6%)
Bad smell	8 (5%)	5 (5%)	3 (6%)

^a^“Other” occupations include security, researcher, and bus driver.

^*∗*^ *p* < 0.05 comparing Akaki-Kality and Yeka using *t*-test for continuous variables and chi-square for categorical variables.

**Table 3:** Creatinine-corrected urine and blood metal concentrations, by subcity—Addis Ababa, Ethiopia, 2011^a^.

	Geometric mean (95% confidence interval)			
	Overall	Akaki-Kality	Yeka	US population^b^
Urine				
Antimony (*µ*g/g creat.)	0.036 (0.031–0.040)	0.036 (0.031–0.042)	0.034 (0.028–0.042)	N/A
Arsenic (*µ*g/g creat.)^*∗*^	5.85 (5.39–6.36)	5.44 (4.99–5.93)	6.83 (5.74–8.13)	8.04 (7.07–9.14)
Barium (*µ*g/g creat.)	2.05 (1.72–2.43)	2.02 (1.62–2.51)	2.12 (1.62–2.77)	1.29 (1.17–1.41)
Cadmium (*µ*g/g creat.)	0.127 (0.112–0.144)	0.133 (0.115–0.156)	0.116 (0.094–0.143)	0.220 (0.204–0.237)
Cesium (*µ*g/g creat.)	1.19 (1.12–1.27)	1.11 (1.02–1.21)	1.39 (1.28–1.50)	4.36 (4.15–4.59)
Chromium (*µ*g/g creat.)	0.412 (0.361–0.470)	0.422 (0.361–0.494)	0.391 (0.306–0.500)	N/A
Cobalt (*µ*g/g creat.)	1.56 (1.38–1.76)	1.64 (1.41–1.89)	1.40 (1.13–1.74)	0.349 (0.330–0.369)
Iodine (*µ*g/L)	55.1 (48.5–62.5)	51.4 (44.8–59.0)	63.4 (49.1–82.0)	N/A
Lead (*µ*g/g creat.)^*∗*^	0.856 (0.779–0.940)	0.944 (0.842–1.06)	0.698 (0.600–0.813)	0.433 (0.402–0.466)
Mercury (*µ*g/g creat.)^*∗*^	0.130 (0.111–0.152)	0.144 (0.122–0.170)	0.103 (0.073–0.145)	0.393 (0.351–0.439)
Molybdenum (*µ*g/g creat.)^*∗*^	111 (99.6–124)	128 (111–147)	83.4 (71.4–97.5)	38.6 (37.1–40.2)
Nickel (*µ*g/g creat.)	8.75 (8.04–9.53)	8.76 (7.91–9.70)	8.73 (7.50–10.2)	N/A
Thallium (*µ*g/g creat.)	0.286 (0.254–0.321)	0.289 (0.248–0.336)	0.279 (0.234–0.332)	0.166 (0.155–0.179)
Tungsten (*µ*g/g creat.)^*∗*^	0.184 (0.159–0.212)	0.216 (0.181–0.259)	0.131 (0.106–0.162)	0.074 (0.067–0.083)
Uranium (*µ*g/g creat.)^*∗*^	0.009 (0.008–0.011)	0.014 (0.012–0.017)	0.004 (0.003–0.004)	0.007 (0.006–0.008)
Blood				
Cadmium (*µ*g/L)	0.233 (0.217–0.249)	0.226 (0.209–0.243)	0.250 (0.217–0.288)	0.337 (0.323–0.353)
Lead (*µ*g/dL)	1.66 (1.53–1.79)	1.65 (1.51–1.79)	1.68 (1.42–1.98)	1.09 (1.03–1.16)
Manganese (*µ*g/L)	9.91 (9.34–10.5)	9.92 (9.20–10.7)	9.87 (9.03–10.8)	9.09 (8.94–9.24)
Mercury (*µ*g/L)	N/A	N/A	N/A	0.863 (0.753–0.990)
Selenium (*µ*g/L)	157 (152–162)	159 (153–166)	153 (146–161)	193 (190–196)

^a^Only metals detected in 5% or more of samples.

^b^The geometric mean and 95% confidence interval among adults (20 years and older) from the 2011-2012 US National Health and Nutrition Examination Survey (NHANES).

^*∗*^ *p* < 0.05 for maximum likelihood estimation comparing Akaki-Kality and Yeka.

N/A: not calculated; proportion of results below limit of detection was too high to provide a valid result.

**Table 4:** Analyte concentrations (mg/L) in drinking water, by subcity—Addis Ababa, Ethiopia, 2011^a^.

	Overall (*n* = 151)			Akaki-Kality (*n* = 101)			Yeka (*n* = 50)		
Analyte	Range	Mean	% >CV	Range	Mean	% >CV	Range	Mean	% >CV
Manganese^*∗*^	<LOD–0.15	<LOD	17%	<LOD–0.150	<LOD	2%	<LOD–0.120	0.028	47%
Nitrates^*∗*^	<LOD–740	1.12	1%	<LOD–5.10	3.48	0%	<LOD–740	<LOD	2%
Lead^*∗*^	<LOD–0.007	<LOD	0%	<LOD–0.007	<LOD	0%	<LOD–0.003	<LOD	0%
Copper	<LOD–0.024	<LOD	0%	<LOD–0.024	<LOD	0%	<LOD	<LOD	0%
Cadmium	<LOD–0.001	<LOD	0%	<LOD–0.001	<LOD	0%	<LOD	<LOD	0%
Arsenic	<LOD–0.004	<LOD	0%	<LOD	<LOD	0%	<LOD–0.004	<LOD	0%
Chromium	<LOD	<LOD	0%	<LOD	<LOD	0%	<LOD	<LOD	0%
Cobalt	<LOD	<LOD	0%	<LOD	<LOD	0%	<LOD	<LOD	0%
Mercury	<LOD	<LOD	0%	<LOD	<LOD	0%	<LOD	<LOD	0%

CV = comparison value (provided in Table 1).

^a^Only metals detected in 5% or more of samples.

^*∗*^ *p* < 0.05 for maximum likelihood estimation comparing Akaki-Kality to Yeka.
